# Protein variability in cerebrospinal fluid and its possible implications for neurological protein biomarker research

**DOI:** 10.1371/journal.pone.0206478

**Published:** 2018-11-29

**Authors:** Lukas M. Schilde, Steffen Kösters, Simone Steinbach, Karin Schork, Martin Eisenacher, Sara Galozzi, Michael Turewicz, Katalin Barkovits, Brit Mollenhauer, Katrin Marcus, Caroline May

**Affiliations:** 1 Medizinisches Proteom-Center, Ruhr-University Bochum, Universitaetsstrasse, Bochum, Germany; 2 Paracelsus-Elena-Klinik, Klinikstraße, Kassel, and University Medical Center Göttingen, Department of Neurology, Göttingen, Germany; Nathan S Kline Institute, UNITED STATES

## Abstract

Cerebrospinal fluid is investigated in biomarker studies for various neurological disorders of the central nervous system due to its proximity to the brain. Currently, only a limited number of biomarkers have been validated in independent studies. The high variability in the protein composition and protein abundance of cerebrospinal fluid between as well as within individuals might be an important reason for this phenomenon. To evaluate this possibility, we investigated the inter- and intraindividual variability in the cerebrospinal fluid proteome globally, with a specific focus on disease biomarkers described in the literature. Cerebrospinal fluid from a longitudinal study group including 12 healthy control subjects was analyzed by label-free quantification (LFQ) via LC-MS/MS. Data were quantified via MaxQuant. Then, the intra- and interindividual variability and the reference change value were calculated for every protein. We identified and quantified 791 proteins, and 216 of these proteins were abundant in all samples and were selected for further analysis. For these proteins, we found an interindividual coefficient of variation of up to 101.5% and an intraindividual coefficient of variation of up to 29.3%. Remarkably, these values were comparably high for both proteins that were published as disease biomarkers and other proteins. Our results support the hypothesis that natural variability greatly impacts cerebrospinal fluid protein biomarkers because high variability can lead to unreliable results. Thus, we suggest controlling the variability of each protein to distinguish between good and bad biomarker candidates, e.g., by utilizing reference change values to improve the process of evaluating potential biomarkers in future studies.

## Introduction

Human cerebrospinal fluid (CSF) is a clear body fluid that is produced by filtration of blood in the choroid *plexus* of the first three brain ventricles. Normally, CSF contains less than five cells per μL. The total protein concentration of CSF varies between 0.2% and 0.5% of the total protein concentration of blood [1]. It is considered that 80% of CSF proteins originate in blood and that CSF proteins are diluted in a molecule-size-dependent concentration gradient [2]. The concentration of blood-derived proteins increases from the ventricles to the cistern to the lumbar CSF. It is assumed that the remaining 20% of CSF proteins are released from the central nervous system (CNS) [2]. The primary role of CSF is to protect the CNS from mechanical shocks [3, 4]. Another important function of CSF is to maintain metabolite clearance from the adult brain by circulation. Moreover, CSF supports the homeostatic balance in the brain and, therewith, normal brain activity [4].

Protein biomarkers are the focus of many studies investigating CNS disorders because the main issue for the clinical diagnosis of such disorders is nonspecific clinical symptomatology [5]. For example, Parkinson’s disease (PD), Alzheimer’s disease (AD), Huntington’s disease (HD), multiple sclerosis (MS) and other neurological disorders usually show many similar symptoms. This similarity often results in a delayed diagnosis or even misdiagnosis [6]. The problem of an ambiguous diagnosis occurs particularly in the early stages of these diseases, when the clinical symptoms (e.g., depression) are not specific to any one of the mentioned disorders [7]. A reliable and definitive diagnosis requires pathological confirmation. However, *in vivo* brain tissue sampling by biopsy to support the clinical diagnosis is often not realizable and results in too many health risks for routine workup in many disorders. Thus, to improve *in vivo* diagnoses and to evaluate disease progression or potential therapeutic effects, new reliable biomarkers are needed [8]. For clinical application, biomarkers need to be easily accessible. In contrast to brain biopsy, CSF is easily and safely accessible by established methods [9]. Several studies already detected CSF alterations decades before the first clinical symptoms appeared [10]. Therefore, many disease and progression biomarkers for neurological disorders were investigated in CSF in the past [11–14]. Regardless of the disease, there is a pronounced lack of reproducibility of these unbiased proteomic studies [12, 15]. For example, for PD, many proteins found in CSF by proteome analysis have been investigated as biomarkers but showed inconclusive or conflicting results [16–18]. These findings also apply with other neurological disorders [14, 19]. One of the factors contributing to this issue could be the high variability of CSF proteins for various reasons [20]. In contrast, AD studies revealed that a combination of the main components of the disease-specific pathological hallmarks are promising biomarkers [21, 22]. However, reliable biomarkers are needed for early diagnosis and upcoming putative neuroprotective trials for all neurological disorders [8, 22, 23].

Multiple studies have previously investigated the CSF proteome in the context of variability and biomarkers in different diseases. For example, in 2005, Hu *et al*. used samples from six subjects obtained within a sampling interval of two weeks. Four subjects were cognitively normal, and two subjects showed indications of very mild dementia. Two-dimensional difference gel electrophoreses (DIGE) was used for differential analysis of protein spots between the sampled time points, followed by tandem mass spectrometry for identification of these proteins [24]. The researchers investigated the intraindividual variability within two weeks between sampling as well as the interindividual variability, but only for the differential protein spots. Furthermore, Hühmer and colleagues summarized the efforts and progress in CSF protein profiling. Evaluating CSF composition studies, the authors published a list of detected CSF proteins that were identified in at least two independent studies of human CSF [15]. A further CSF proteome study was performed by a Canadian research group in 2016. The main purpose of this study was to identify brain-related proteins in CSF that are suitable for the development of diagnostic assays. The authors found 78 brain-related proteins in CSF of at least 4 of 6 healthy individuals [25]. Another study analyzed the interindividual variability in CSF protein abundances in samples of 9 patients undergoing routine, nonneurological surgical procedures [20]. The researchers presumed that an understanding of the biological variation in CSF proteins healthy individuals is essential for reliable interpretation of studies for neurological disorder biomarkers [20]. In 2018, Trombetta *et al*. implemented a fit-for-purpose modeled approach to qualify a broad selection of commercially available immunoassays [26]. Therefore, paired baseline and eight-week CSF samples from twenty participants with mild cognitive impairment or mild dementia due to AD were used [26]. Trombetta *et al*. demonstrated consistent sensitivity, reliability and biotemporal stability of different immunoassays for 32 different CSF analytes i.a. by calculating the coefficients of variation (CVs) [26].

The aim of this study was to investigate the implications of the high variability in CSF protein composition and protein abundance in healthy control subjects for biomarker discovery studies. It is known that the variability is influenced by the analytical variability as well as the biological variability [27, 28]. In addition to standard deviation, variance and CV, a measure of variability is the reference change value. This value represents the change in the abundance of a protein (e.g., a potential biomarker) that is necessary for the change to be considered larger than expected after taking into account biological and technical variance [29]. Via unbiased LFQ via LC-MS/MS, we investigated CSF samples obtained from a longitudinal study group of 12 healthy control subjects (12 samples for every time point: 0 months, T_0_; 24 months, T_24_; 48 months, T_48_) and determined the intra- and interindividual variability, as well as the reference change value, for each protein.

## Materials and methods

### Ethical statement

The ethics committee of the Physician's Board Hessen, Germany (approval no. FF89/2008) approved this study, which is registered at the German Register for Clinical Trials (DRKS00000540) according to the WHO Trial Registration Data Set. All participants provided written, fully informed consent to participate in this study. The relevant documents relating to this process are archived at Paracelsus-Elena-Klinik in Kassel, Germany. Only the anonymous data and materials from the participants were provided to the scientists carrying out the research. The data concerning this study were stored separately from the hospital charts of the patients.

### Subjects and samples

This CSF core proteome study has a longitudinal design with 12 human subjects evaluated at three different time points. The subjects were selected as previously described by Mollenhauer *et al*. [18]. These 12 healthy subjects are a representative group of 90 neurologically unimpaired healthy subjects who are participating in the longitudinal DeNoPa study (de Novo Parkinson study), which is a prospective, single center study performed as described by Mollenhauer *et al*. [18]. All subjects (volunteer CSF donors) were recruited at Paracelsus-Elena-Klinik in Kassel, Germany. The subjects were not eligible for the study if they demonstrated cognitive impairment (Mini-Mental State Examination score < 27) or any other neurological disease. A neurologist evaluated qualified subjects according to a standardized assessment protocol, including a neurological examination and MRI, among tests (S1 Table). This examination was repeated at every time point (T_0_, T_24_, T_48_) for every subject. No subject showed symptoms of any neurological disease. Further, routine laboratory blood analysis was carried out. Blood was collected with BD Vacutainer system tubes (BD, Franklin Lakes, NJ, USA) by venous puncture and processed according to published standard operating procedures (SOPs) between 7 and 9 a.m. after 12 hours fasting [18]. Aliquots were stored at -80 °C within 30 min following the venous puncture.

### Characteristics of the study group

The age and gender characteristics of our study group are shown in Table 1. The median age of human subjects was 65 at the beginning of the study, with 39.1% of the subjects being female. The mean time between each of the follow-up assessments (T_0_ (first), T_24_ and T_48_) was two years. Routine blood and CSF laboratory analyses were performed to exclude severe diseases that can affect CSF. Furthermore, blood contamination of CSF was clinically assessed by the erythrocyte count. In some samples, slight contamination from blood was found (0 to 64 erythrocytes, median: 0) but were negligible, as described by Reiber [1]. Otherwise, no significant deviations related to default values were revealed in the routine laboratory analysis of the CSF. With all the tests and information, the selected study group covered all characteristics necessary for our study.

**Table 1:** Gender, age and neuropsychological characteristics of the representative study group.

	Standard value	Study group						
		N	f*	m*	Mean	SD*	CV*	Median
Age [years]	40–85	12	5	7	69.1	4.7	6.7%	69.0
NMS Quest total [points]	rating scale	12	5	7	4.3	2.9	67.7%	4.0
NMS Quest sum [points]	rating scale	12	5	7	0.1	0.1	68.4%	0.1
UPDRS total [points]	rating scale	12	5	7	2.2	2.2	99.6%	2.0
MDS UPDRS total [points]	rating scale	12	5	7	6.3	5.0	79.8%	5.5
BDI total [points]	0–8	12	5	7	5.0	4.5	90.2%	3.1
MMSE total [points]	24–30	12	5	7	28.3	1.5	5.3%	29.0
Clock test: [points]	<2	12	5	7	1.3	0.6	43.3%	1.0

Of the 90 human subjects participating in the DeNoPa study twelve were selected for this CSF study. The representative study group analyzed in the present study reflects the total study group in terms of gender frequencies, age distribution as well as neuropsychological characteristics.

*SD = standard deviation, CV = coefficent of variation, f = female, m = male.

### CSF sampling for routine laboratory analysis and mass spectrometric analysis

CSF was obtained from the subarachnoid space of the lumbar spinal cord by lumbar puncture. Sampling was performed as described by Mollenhauer *et al*. [18]. Routine clinical variables were determined using established routine protocols (Table 2 and S2 Table). The CSF was centrifuged at 2,500 x g for 10 min at room temperature, and the supernatant was collected for further analysis. The time between obtaining the sample and the centrifugation step was under 30 min. Within 30 min, the samples were stored in aliquots at -80 °C until further analysis.

**Table 2:** CSF and routine laboratory analysis for the representative study group analyzed in the present study.

	Standard value	Study group				
		N	Mean	SD*	CV*	Median
Proteins [mg/L]	200–400	12	424.6	106.9	25.2%	413.5
White blood cell count [cells/mm3]	0–4	12	0.4	0.6	151.9%	0.0
Erythrocyte count [cells/mm3]	0–10	12	4.9	13.3	271.8%	0.0
CSF albumin [mg/L]	0.0–350.0	12	276.2	77.3	28.0%	255.5
Serum albumin [g/L]	35.00–55.00	12	42.7	2.6	6.1%	42.8
Albumin Quotient [ratio]	< 8	12	6.5	1.8	27.4%	6.2
CSF IgG [mg/L]	10.0–40.0	12	29.4	8.0	27.4%	30.5
Serum IgG [g/L]	8.0–18.0	12	10.0	1.8	18.2%	9.7
IgG Quotient [ratio]	~ 2,3	12	3.0	0.9	29.4%	2.8
CSF IgA [mg/L]	1.5–6.0	12	5.3	2.9	55.3%	4.6
Serum IgA [g/L]	0.9–4.5	12	3.4	1.6	46.2%	3.1
IgA Quotient [ratio]	~ 1,3	12	1.5	0.6	39.9%	1.4
CSF IgM [mg/L]	0.0–1.0	12	0.2	0.1	37.5%	0.2
Serum IgM [g/L]	0.6–2.5	12	1.0	0.5	48.0%	0.8
IgM Quotient [ratio]	~ 0.3	12	0.3	0.3	82.7%	0.2

Clinical CSF parameters (e.g., red and white blood cell count and protein concentrations) were determined to ensure a healthy study group. In addition CSF/serum quotients were determined.

*SD = standard deviation, CV = coefficent of variation.

### CSF sample preparation for mass spectrometric analysis

Before performing mass spectrometric analysis, protein digestion of the CSF samples was performed according to the protocol published by Stoop *et al*. with slight modifications [20]. In short, CSF samples (50 μL) were incubated 1:1 with 0.2% (s/v) RapiGest SF Surfactant (Waters Corporation, Milford, MA, USA) (in 50 mM ammonium bicarbonate). Proteins were reduced with 5 mM dithiothreitol (final concentration) at 60 °C for 30 min. Next, iodoacetamide was added for alkylation (final concentration of 15 mM), and the samples were incubated at room temperature for 30 min in the dark. Tryptic digestion was performed at 37 °C overnight (16 hours) at a 1:50 (w/w) trypsin-to-protein ratio. Digestion was stopped by adding 25% (v/v) trifluoroacetic acid (TFA) for a final concentration of 0.5% (v/v). After incubation at 37 °C for 40 min, the samples were centrifuged (Centrifuge 5417R, Rotor: FC45-30-11, Eppendorf AG, Hamburg, Germany) at 17,000 x g and 4 °C for 10 min. Clear supernatants of the samples were transferred into new reaction tubes and dried with a rotational vacuum concentrator (SpeedDry RVC 2–25 CDplus, Martin Christ Gefriertrocknungsanlagen GmbH, Osterode, Germany). Finally, samples were resuspended in 0.1% (v/v) TFA. The peptide concentration was measured by amino acid analysis, as described by Plum *et al*. [30].

### Mass spectrometric analysis

Protein digests were analyzed on a nanoHPLC system (UltiMate 3000, Dionex, Idstein, Germany) coupled on-line to a quadrupole orbitrap mass spectrometer (Q Exactive, Thermo Fisher Scientific, Bremen, Germany). Samples were injected into the nanoHPLC system by an autosampler and were loaded on a C18 trap column (PepMap100 C18, 100 μm ID x 2 cm, particle size 5 μm and pore size 100 Å; Thermo Scientific, Rockford, IL, USA) using 0.1% (v/v) TFA and a flow rate of 30 μL/min. After sample loading, the trap column was switched to an analytical C18 column (PepMap C18, 75 μm x 50 cm, particle size 5 μm and pore size 100 Å; Thermo Scientific, Rockford, IL, USA). For peptide separation, the following solvent system was used: buffer A: 0.1% (v/v) formic acid; buffer B: 84% (v/v) acetonitrile, 0.1% (v/v) formic acid. A linear gradient of 4–50% buffer B was carried out for 180 min at a flow rate of 400 nL/min, followed by a washing step with 95% B for 5 min and an equilibration step with 5% B for 5 min. The column oven temperature was set to 60 °C. Ionization took place in a nano electrospray ionization source (ESI), and mass spectrometric analysis was performed in data-dependent scan mode. For MS/MS analysis, full MS spectra were scanned in the range from 350–1,400 m/z with a resolution of 70,000 at 200 m/z (automatic gain control (AGC) target 2e6, 80 ms maximum injection time). The spray voltage was set to 1,600 V, and the capillary temperature was set to 250 °C. For internal recalibrations, the lock mass polydimethylcyclosiloxane (m/z: 445.120) was used. The 10 ions with the highest intensities were selected for higher energy collision-induced dissociation (HCD) fragmentation. MS/MS fragments were generated with a 27% normalized collision energy, an isolation window of 2.2 m/z and a fixed first mass of 130 m/z. An orbitrap analyzer with a resolution of 35,000 at 200 m/z (AGC 5e5, maximum injection time 120 ms) was used for fragment analysis. For the assessment of the analytical variability, seven replicates of pooled samples were measured.

### Protein quantification

Mass spectrometric data were analyzed with the intensity-based quantification software MaxQuant (version 1.5.3.12) [31]. MS/MS spectra were searched against the UniProt/Swiss-Prot [32] human proteome database (UniProtKB/Swiss-Prot UniProt release 2017_01; downloaded 2017-01-26; number of entries 553,474) using the search engine Andromeda [33]; the search included 262 common contaminants and concatenated with the reversed versions of all sequences [34]. The precursor and fragment ion mass tolerance were set to 5 ppm and 20 ppm, respectively. The enzyme specificity was set to trypsin, and two missed cleavages were allowed. The minimum peptide length was set to 7 amino acids. Cysteine carbamidomethylation was set as fixed, and methionine oxidation and N-terminal acetylation were set as variable modifications. A maximum of 5 modifications per peptide was set. For both peptide spectrum matches (PSMs) and protein level, the false discovery rate (FDR) was set to 1%. For the calculation of the protein abundances, label-free quantification (LFQ, [35]) was performed with an LFQ minimum ratio count of two. Normalized LFQ intensities were used for further data analysis.

### Mathematical calculation of the variation in protein abundance

For each protein, its CV is defined as the ratio of the standard deviation (s) to the mean ($\bar{x}$) in %:

$$CV=s/\bar{x}*100$$ (1)

In this paper, several types of CVs are used. First, to assess the analytical variability or inaccuracy, CVa can be calculated using the standard deviation and mean of repeated measurements of the same sample. To this end, in this paper, 4 technical replicates were used. Second, for each subject, protein abundances were measured at three different time points (i.e., T_0_, T_24_ and T_48_), and for each protein, a CV for the three abundances was calculated. The median of these CVs across all samples is called CV_t_ and represents the intraindividual variability over time. Finally, to obtain the measured interindividual CV between different subjects (CV_g_), the mean and the standard deviation of the protein abundances across all samples at T_0_ are used in formula (1) to obtain CV_g_.

The reference change value (RCV) describes the change in the protein abundance (e.g., a potential protein biomarker) in % that is added to the original abundance (i.e., 100%) and is necessary to reflect an ‘“unexpectedly high” change [29], i.e., a change that is unlikely to occur based only on the analytical, inter- or intraindividual variability and might be biologically relevant (e.g., a potential protein biomarker). The original formula is given in (2):

$$RCV=1.96*\sqrt{2}*\sqrt{{{CV}_{a}}^{2}+{{CV}_{i}}^{2}}$$ (2)

with CV_i_ standing for the intraindividual biological CV given by

$${CV}_{i}=\sqrt{{{CV}_{t}}^{2}-{{CV}_{a}}^{2}}$$ (3)

The factor 1.96 corresponds to a 95% confidence level assuming a normal distribution. By inserting formula (3) into formula (2), CV_a_^2^ is canceled out, and the square root vanishes. Therefore, the formula for the RCV can be simplified to

$${RCV}_{t}=1.96*\sqrt{2}*{CV}_{t}$$ (4)

This RCV is called RCV_t_ because it uses CV_t_ and applies to the intraindividual variability. We modify the definition for RCV_g_ by exchanging CV_t_ for CV_g_ in order to reflect “unexpectedly high” changes between different individuals:

$${RCV}_{g}=1.96*\sqrt{2}*{CV}_{g}$$ (5)

Generally, the RCV is always larger than zero, and in order to compare the RCV to fold changes, the original abundance (i.e., 100%) needs to be added so that (*RCV* + 100%)/100% has to be considered. Then, whether a fold change is unexpectedly high can be assessed. For a CV_g_ value of 50%, the following RCV_g_ can be calculated:

$${RCV}_{g}=1.96*\sqrt{2}*50\%$$ (6)

$${RCV}_{g}=139\%$$ (7)

Consequently, for this example, the fold change should be larger than 2.39 to reflect an “unexpectedly high” change in protein abundance.

## Results

### Basic technical information of LC-MS/MS runs

**Fig 1:**
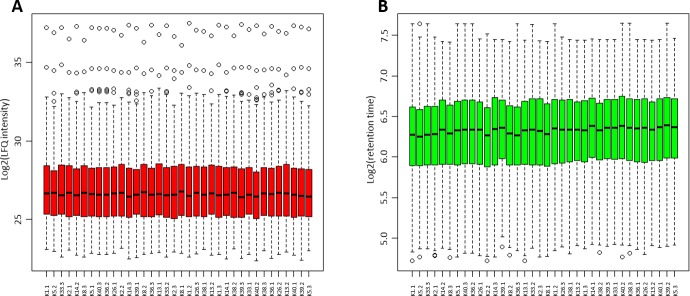
Box plots of LFQ intensities and peptide retention times. Log2 of the LFQ intensities of all proteins, which were quantified in 100% of the samples, are shown in A (red box plots). The data were not further normalized or transformed once the values from MaxQuant were obtained. **B** shows the log2 of peptide retention times for every LC-MS/MS run (green box plots).

In quantitative mass spectrometry, accuracy is highly important. Therefore, in Fig 1, box plots of the LFQ intensities and peptide retention times plotted in the order in which the samples were run are shown. Furthermore, we investigated the analytical variation in our method (CV_a_)_._ To calculate the analytical variability CV_a_, pooled samples were measured four times during the whole measuring sequence for the 36 individual samples. The resulting average CV_a_ was 8.49%.

### Global description of proteins identified in CSF

**Fig 2:**
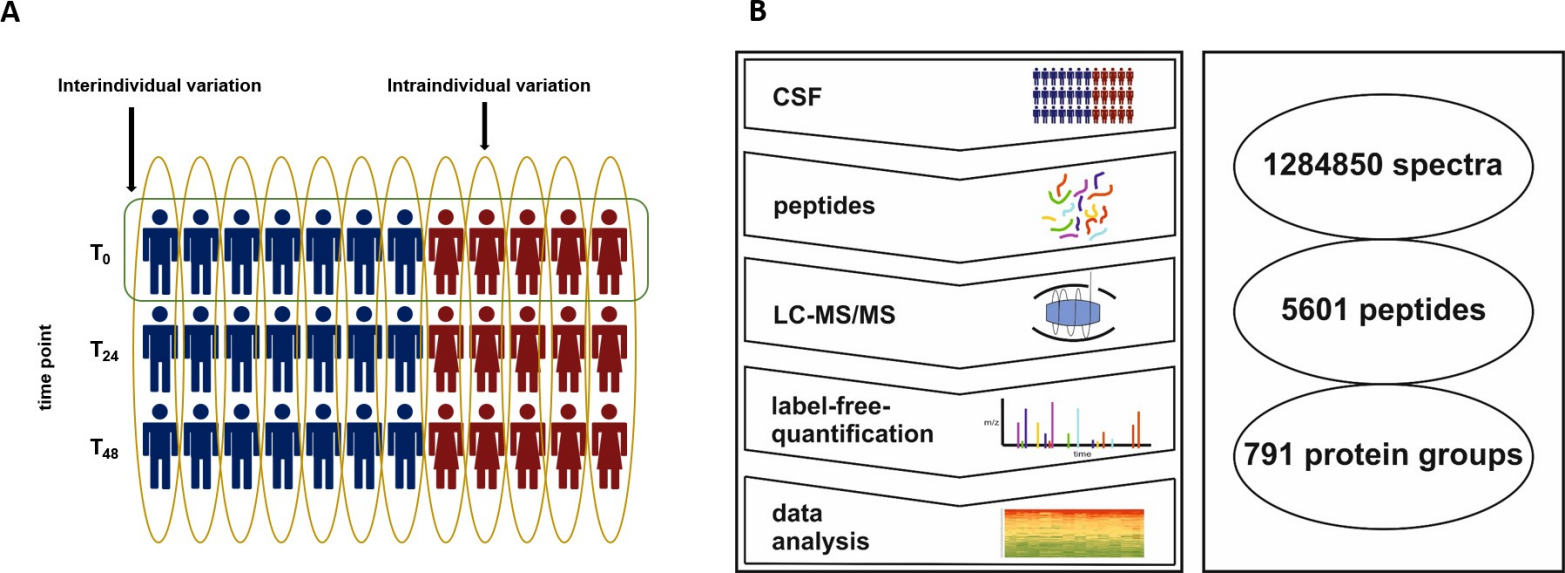
Study overview. **A**: Two variables, time point (T_0_, T_24_ and T_48_) and human subject (red figures represent female subjects, and blue figures represent male subjects), can characterize every sample in this study, and for every human subject, three samples were obtained. In this study, the inter- and intraindividual variability of the CSF were analyzed. Hereby, the intraindividual variability is defined as the variation within one human subject between the three time points (yellow circle), and the interindividual variability is defined as the variation between all subjects at T_0_ (green circle). **B**: The workflow included CSF sampling, sample preparation, tryptic digestion, LC-MS/MS with LFQ and data analysis. We identified 1,284,850 spectra, which resulted in 5601 peptides and 791 protein groups.

We identified a total of 5601 peptides across all 12 analyzed samples from neurologically healthy individuals (Fig 2), and the three time points, T_0_, T_24_ and T_48_, together with 36 samples, were analyzed. These peptides were matched to 791 protein groups hereinafter briefly referred to as “proteins” by MaxQuant’s protein inference (e.g., see [36] regarding the relevance of protein inference). The selection criterion used to obtain this number of proteins was at least one identified unique peptide per protein across all samples. To investigate the variability, only 223 proteins that were found in all 36 samples were used (S1 File). Then, to increase the significance of the identified proteins, the selection criterion was set to two unique peptides per protein. With this choice, the number of proteins decreased from 791 to 610 proteins (S2 File) and from 223 to 216 (S3 File). In the following, this resulting protein list (S3 File) will be referred to as the “CSF core proteome”.

### High variability of protein abundance in the CSF core proteome

**Fig 3:**
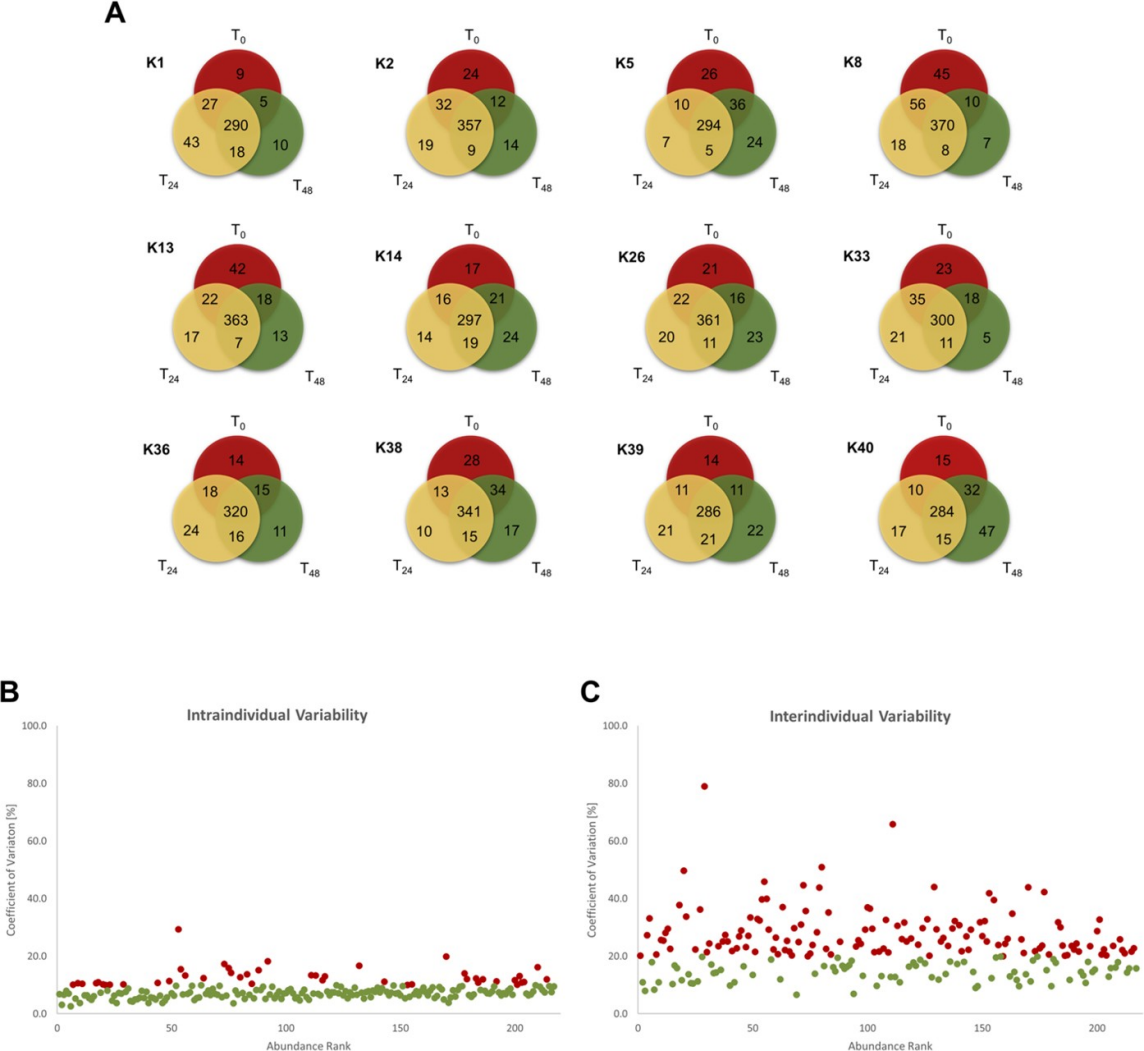
Intra- and interindividual variability. **A**: This figure provides twelve Venn diagrams, one for each human subject. Only proteins that were found with at least 2 unique peptides in one of three samples were counted. The three time points are represented by different colors. Proteins that were identified in all three samples are shown in the middle overlap. Each overlap between two circles gives the number of proteins that were identified in these two samples. **B**: CV_t_ values were calculated for all 216 proteins of the CSF core proteome and were plotted against their abundance rank. Proteins with CV_t_ > 10% are colored in red, and those with CV_t_ < 10% are colored green. **C**: CV_g_ values were calculated for all 216 proteins of the CSF core proteome and were plotted against their abundance rank. Proteins with CV_g_ > 20% are colored red, and those with CV_g_ < 20% are colored green.

To assess intraindividual variability, first, the protein overlap of samples from different time points (T_0_, T_24_ and T_48_) for each individual human subject was compared. For visualization of the intraindividual overlaps, Venn diagrams were generated (Fig 3A). Within one human subject, between 284 and 370 proteins were found at all three time points. Furthermore, between 5 and 47 proteins were identified in only one subject at a particular time point. There was no subject with a 100% overlap of identified proteins at all three time points.

**Fig 4:**
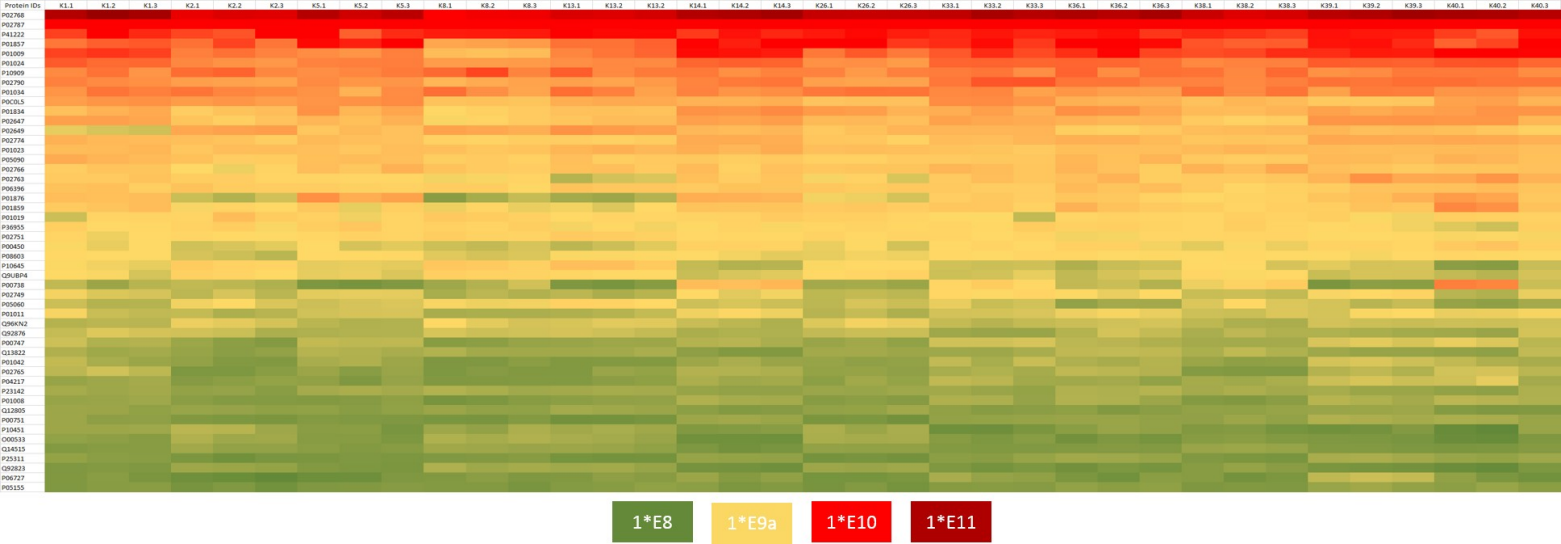
LFQ intensity-based heatmap of the top 50 proteins. LFQ intensity-based heatmap of the top 50 proteins. Top 50 proteins were defined by their mean LFQ intensity of all samples. In each column, the LFQ intensity for one sample is visualized. The color range of LFQ intensity extends from 1e8 (green) over 1e9 (yellow) and 1e10 (red) to 1e11 (dark red).

Additionally, to assess the intra- and interindividual variability, CV_t_ and CV_g_ were calculated. The calculated CV_t_ values were between 2.6% and 29.3%, with a mean of 8.2% (S2 File). The determined CV_g_ was between 9.3% and 101.5%, with a mean of 25.7% (S1 File). To visualize the intra- and interindividual variability, the CV_t_ and CV_g_ values of the CSF core proteins were plotted against their abundance rank (Fig 3B and 3C), and the top 50 proteins of the CSF core proteome showing the highest intensities were plotted in a heatmap to give a broad sense of the variability within and between individuals (Fig 4). Here, the protein with the highest mean LFQ intensity (1.43e11) was serum albumin (P02678; ALB), followed by serotransferrin (P02787; TF; mean LFQ intensity: 2.47e10), prostaglandin-H2 D-isomerase (P4122; PTGDS; mean LFQ intensity: 8.86e9) and Ig gamma-1 chain C region (P01857; IGHG1; mean LFQ intensity: 7.68e9). The CV_g_ values from the top 50 abundant proteins ranged from 9.8% (prostaglandin-H2 D-isomerase) to 101.5% (haptoglobin). The CV_t_ values varied between 2.6% (complement C3) and 11.3% (apolipoprotein A-IV) (S3 Table).

We determined RCV_t_ and RCV_g_ for all proteins of the CSF core proteome. The obtained RCV_t_ values ranged from 7.2% to 81.3% (S3 File), with a mean of 25.7%. The RVC_g_ values varied between 19.2% and 281.4%, with a mean of 71.2%. The highest RCV_g_ was calculated for haptoglobin, and the highest RCV_t_ was calculated for beta-2-microglobulin.

In conclusion, there is a wide range of variability in protein abundance in the CSF core proteome between subjects as well as within one subject. This variability is also indicated by the broad range of RCVs. This high variability might have been attributed to a false identification of potential protein biomarkers. To evaluate this assumption, we looked more closely at specific proteins within our CSF core proteome that were proposed in previous studies as potential protein biomarkers for different neurological diseases.

### Protein biomarkers often identified in various diseases

Several proteins have been proposed as biomarker candidates for neurological disorders of the CNS. However, these proteins exhibited partially contradictory results. In this context, we generated a list of CSF protein biomarker candidates by searching the literature regarding potential CSF biomarkers with a particular focus on the neurological diseases AD, PD, MS and HD. Table 3 presents 20 proteins of the CSF core proteome that were mentioned in different publications as potential biomarkers for several diseases and were detected by mass spectrometric approaches. The CV and RCV values of these proteins were assessed. Generally, low CVs and RCVs might hint at the possibility that a protein could be a suitable biomarker. However, these proposed biomarkers show a wide range of variability.

**Table 3:** Protein biomarker candidates with their CVs and RCVs.

Protein IDs	Gene name	Protein name	CV_t_ / CV_g_ [%]	RCV_t_ / RCV_g_ [%]	PD/PDD	AD	HD	MS	Other diseases
**P00738**	HP	Haptoglobin	10.2 / 101.5	28.2 / 281.4	[37]	[37, 38]		[39]	
**P02675**	FGB	Fibrinogen beta chain	13.4/ 45.5	37.0 / 126.0	[37]		[40]		
**P02652**	APOA2	Apolipoprotein A-II	17.3 / 46.6	48.0 / 129.3	[37]			[41]	[42]
**P06727**	APOA4	Apolipoprotein A-IV	11.3 / 33.1	31.4 / 91.7				[39, 41]	
**P61769**	B2M	Beta-2-microglobulin	29.3 / 52.5	81.3 / 145.5	[37]	[43]			
**P02649**	APOE	Apolipoprotein E	6.5 / 31.8	17.9 / 88.2		[44, 45]		[46]	[42, 44]
**P02753**	RBP4	Retinol-binding protein 4	15.1 / 27.7	42.0 / 76.9	[37]		[40]	[41]	
**P25311**	AZGP1	Zinc-alpha-2-glycoprotein	4.5 / 23.5	12.6 / 65.1	[37]	[47]			
**P02774**	GC	Vitamin D-binding protein	5.5 / 22.6	15.2 / 62.7		[44]		[39, 41]	[44]
**P02749**	APOH	Beta-2-glycoprotein 1	7.7 / 23.6	21.2 / 65.5	[37]		[40]	[41]	
**P00441**	SOD1	Superoxide dismutase [Cu-Zn]	15.9 / 32.3	44.1 / 89.6		[37]			[37]
**P02768**	ALB	Serum albumin	6.9 / 21.0	19.0 / 58.3		[48]		[41]	
**P01024**	C3	Complement C3	2.6 / 19.0	7.2 / 52.6			[40]	[41, 49, 50]	
**P05090**	APOD	Apolipoprotein D	6.1 / 19.9	17.0 / 55.1		[37]		[41, 49]	
**P36222**	CHI3L1	Chitinase-3-like protein 1	5.2 / 30.2	14.5 / 83.8		[44, 45]		[39]	[44]
**P02766**	TTR	Transthyretin	10.7 / 19.0	29.5 / 52.8		[44]			[37]
**Q13822**	ENPP2	Extonucleotide pyrophosphatase	5.6 / 14.7	15.5 / 40.9		[44]	[40]		[37]
**Q92876**	KLK6	Kallikrein-6	5.0 / 16.4	13.7 / 45.5	[37]	[37]		[41, 49]	
**P01034**	CST3	Cystatin-C	10.6 / 15.4	29.4 / 42.7	[37]	[38, 44]		[41, 49]	[44]
**P10909**	CLU	Clusterin	10.2 / 12.8	28.2 / 35.4	[38]	[44]		[41, 49]	[44]

20 protein biomarker candidates with their respective intra- and interindividual coefficient of variation (CV_t_ and CV_g_) and reference change values (RCV_t_ and RCV_g_) are listed as well as the studies in which they have been proposed as biomarker candidate.

## Discussion

Generally, the suitability of a molecule as a biomarker depends on its biological variability [27, 29]; thus, we presume that this condition also applies to protein biomarkers in neurological diseases. Unfortunately, the current knowledge about protein variability in CSF is insufficient, although there are some CSF protein variability studies. In 2010, Schutzer *et al*. investigated a pair of individual CSF samples obtained four weeks apart from 10 healthy persons by LC-MS/MS. The authors concluded that there might be general variability, which is relatively limited in a single individual over a short time [51]. Perrin *et al*. went further in 2013. Based on their LC-MS/MS study of two aliquots of CSF samples from 6 cognitively normal individuals, the researchers suggest that interindividual variability has strong implications for the potential of a protein to serve as a biomarker [52]. Unfortunately, both studies have limitations because neither of these studies performed an investigation over a long period. Furthermore, Perrin *et al*. focused more on the technical process for CSF biomarker discovery [52]. In this context, the purpose and scientific contribution of this study was to investigate the variability of the CSF proteome in healthy control subjects in order to determine its impact on biomarker discovery studies and provide a statistical strategy to assess protein variability in future studies.

### Updating the basic protein composition of CSF

In the last two decades, several studies investigated and expanded our knowledge of the CSF proteome [15, 20, 25]. By comparing our results to those of the three most relevant publications, we could confirm the previous findings and further expand them.

First, the proteins detected in the CSF core proteome were compared to the proteins in Table 3 of Hühmer *et al*.; the latter proteins included 90 proteins that were measured in two or more independent compositional CSF studies with gel-based analysis or gel-free LC/MS approaches [15]. In the CSF core proteome, 70 of these 90 proteins were also detected. Furthermore, 2 proteins listed by Hühmer *et al*. were obsolete in the UniProt database (P01028 and P62988; gene symbols n.a.). Of the 18 missing proteins, 8 were not identified in the CSF core proteome: O14791 (APOL1), P09211 (GSTP1), P35542 (SAA4), P60709 (ACTB), P68871 (HBB), P69905 (HBA1), Q06830 (PRDX1) and Q9NQX5 (NPDC1). Ten could not be identified in our complete data set of 791 proteins.

Second, the proteins identified in the CSF core proteome were compared to a total of 126 CSF proteins identified by Stoop *et al*. [20]. The results showed that 114 proteins of these proteins were also detected in the CSF core proteome. However, 11 of the missing 12 proteins were identified in our data set but not in 100% of our samples. One protein was identified as a contamination, and one protein was obsolete in the UniProt database (P62988; gene symbol n.a.).

Third, the proteins detected in the CSF core proteome were compared to 78 brain tissue-enriched and group-specific candidates, as provided in Supplemental Table 5 (SM5) within Begcevic *et al*. [25]. The results showed that 20 of these 78 proteins were identified in our CSF core proteome. However, 38 of the missing proteins were identified in our data set but again not in 100% of our samples.

In summary, our results are in accordance with the literature. Thus, we propose a summarizing CSF core proteome of 244 proteins in healthy human individuals (S4 File). This list is based on proteins identified by our group, Stopp *et al*., and Hühmer *et al*.

### General view–CV_a_, CV_t_ and CV_g_

First, we determined the analytical variability by measuring pooled samples during the whole measuring sequence of the 36 samples. It is recommended to assess analytical variability because it may influence statistical values, such as fold changes or *p*-values, may distort other statistical methods, such as subgroup detection [53], and should be as small as possible. Moreover, CV_a_ should be definitely lower than CV_t_ or CV_g_ to enable the possibility of detecting differences in protein abundance between study groups. As reported above, we determined an average CV_a_ of 8.49%. Generally, high CV_a_ values should be critically reviewed because imprecision in the analytical workflow may be responsible for high variability in protein abundance. Therefore, we assume that CV_t_ and CV_g_ values higher than 8.49% were caused by additional biological variation.

As reported above, for the CSF core proteome, CV_g_ values up to 101.5% were observed. These results are in line with the findings of Stoop *et al*., who assessed the interindividual variability of 126 CSF proteins [30] and concluded that brain-specific proteins are more likely to have a higher CV_g_ than blood proteins [30]. However, the criteria for whether a protein is blood or brain specific are not described in detail. In our study, blood-specific proteins also exhibited high CVg values (e.g., apolipoprotein a-II, fibrinogen beta chain and Ig alpha-2 chain C region) (S1 File From our point of view, the origin of a protein is not the determining factor for its interindividual variability (CV_g_). The range of CV_g_ for the 216 proteins measured in our study is relatively wide (9.3% to 101.5%). We checked our data to determine whether processed peptides are responsible for some high CVs. After comparison of single peptides between the samples, we found no indication that there are possible processed peptides or at least small peptides that behave differently across time or between subjects. Furthermore, we examined whether age influences the variability. We did not find any relationship between these two variables (see S1 Fig). Thus, we anticipate that interindividual variability negatively influences the outcome of biomarker discovery studies, indicating that CV_g_ should be taken into account when rating changes in protein abundance between study groups. For some proteins with high CV_g_ values, only substantial changes might be relevant.

As reported above, in our data set, we observed that intraindividual variability (CV_t_) ranged between 2.6% and 29.3%. Our results show a certain degree of variability within an individual over a four-year period. This finding indicates that CSF proteins vary in their abundance within one individual to different degrees.

In summary, CV_t_ is usually lower than CV_g_,_,_ which is in line with Fraser *et al*. [27], and the variability of a given protein is mainly influenced by its CV_g_ value. Consequently, a biomarker with low CV_t_ and CV_g_ values may be useful for reflecting changes in clinical status. Ideally, the variability in both the disease group and the control group would be low, with a large difference between the means of the groups. A higher variability leads to a decreased power to find changes in protein abundance between healthy and diseased subjects. As a consequence, the results for potential biomarkers with higher CV_g_ values are likely to be less reproducible in further studies. Furthermore, large sample sizes are needed due to the high variability, which might not be feasible for rare diseases. To evaluate the relevance of changes, we calculated the reference change value for each protein within subjects (RCV_t_) and between subjects (RCV_g_) to assess whether changes in protein abundances are expected or unexpected. As reported above, in our CSF core proteome, we found RCV_t_ values up to 81.3% and RCV_g_ values up to 281.4%. Due to these high abundance variabilities, for CSF proteome studies, we recommend calculating the RCV_t_ and RCV_g_ values for a meaningful interpretation of detected protein changes [24]. Moreover, it is possible to estimate a fold change threshold in order to decide whether a given fold change is expectedly high after taking into account its specific natural variability or whether it is unexpectedly high and a potential biomarker candidate. To this end, we suggest calculating RCV_g_ for each quantified protein and computing the “maximum fold change assessment value”, ${FC}_{max}^{*}$, as follows:

$${FC}_{max}=max\{A/B,B/A\}$$ (8)

$$where\;{FC}_{max}=maximum\;fold\;change,A\;and\;B=mean\;protein\;abundances$$

$${FC}_{max}^{*}={FC}_{max}-\left(\frac{RCVg}{100}\right)-1$$ (9)

$$where\;{FC}_{max}^{*}=maximum\;fold\;change\;assessment\;value;\;RCVg=interindividual\;reference\;change\;value$$

We interpret ${FC}_{max}^{*}$ as an unexpectedly high change if ${FC}_{max}^{*}>0$. Otherwise, the change in protein abundance is probably mainly caused by protein-specific natural variability.

In progression marker or clinical improvement studies, RCV_t_ should be used instead of RCV_g_ to compute the above assessment value because in these cases, the intraindividual variability is more important.

### Closer look–Focus on proposed protein biomarkers

To evaluate whether high CV_t_, CV_g_, and RCV values might be a cause for the lack of reproducibility of CSF protein biomarkers in neurological diseases (PD, AD, MS, HD and others), we focused on published data from the last few years and generated a list of 20 proposed CSF protein biomarkers. These proteins were detected in studies with mass spectrometric approaches and were found in our CSF core proteome. As reported above, the maximum CV_t_ value for these 20 proteins was 29.3%. The protein with the highest CV_g_ was haptoglobin (CV_g_: 101.5%), and clusterin had the lowest value (CV_g_: 12.8%). Compared to the CV_t_ values, the CV_g_ values were usually higher. These results indicate that the published biomarkers have a certain degree of variability, which is different for each protein. To examine whether the changes detected in these studies are expectedly high, we calculated RCV_g_ for each of the 20 proteins. The resulting RCV_g_ values ranged from 35.4% to 281.4%. Most of the proposed biomarker candidates had an RCV_g_ value above 50% in our study. This value would imply ${FC}_{max}^{*}$ = 0.5 in the worst case of *FC*_*max*_ = 1. Consequently, a fold change of at least 1.5 is needed for an ${FC}_{max}^{*}$ value above 0, representing an unexpectedly high change in abundance. As another example, haptoglobin showed an RCV_g_ value of 281.4% in our study, which would indicate an ${FC}_{max}^{*}$ value of 2.814 if *FC*_*max*_ = 1. Thus, the fold change in haptoglobin must be larger than 3.814; otherwise, there is no unexpectedly high change in protein abundance. On the other hand, proteins that exhibit a low RCV_g_ (i.e., under 100%) might be better suited as biomarker candidates. As a consequence, some of the 20 proposed protein biomarkers might not have a relevant change in protein abundance between the study groups. Meijers *et al*. showed that similar assumptions are true for established protein markers measured in routine laboratory tests in the context of chronic heart failure prognosis [29].

## Strengths and weaknesses

In our study, we assessed the variability in the CSF proteome within neurologically healthy control subjects over a period of four years. Our identified CSF proteins correspond to the literature [15, 20]. The concordance with Hühmer *et al*. and Stoop *et al*. ensures the good reproducibility, high confidence, and transferability of our defined CSF core proteome. This proteome may serve as a reference list for future CSF proteome studies. To our knowledge, this was the first study to investigate the variability in the CSF proteome by employing bottom-up mass spectrometry in order to identify and quantify as many CSF proteins as possible and by using the RCV for fold change assessment. A targeted MS approach would have allowed us to focus on single biomarker candidates. However, we preferred to gain a broader picture and provide data for future biomarker studies. To this end, we used a label-free MS approach. Owing to our study design, we focused on the variability in protein abundance in neurologically unimpaired healthy control subjects. Further studies must evaluate whether the variability within diseased groups might be comparable to our data. It can be assumed that the variability is strongly dependent on the disease. Furthermore, we suggest investigating whether the variability assessed over shorter periods is similar to our results. However, CSF sampling within short periods can increase the risk of adverse side effects for the subjects. We chose longer periods on the order of years because the long-term behavior of biomarkers is clinically important. These biomarkers should reflect the course of the disease. For an alternative to the RCV for evaluating “unexpectedly high” changes in protein abundance, it might also be reasonable to conduct a receiver operating characteristic (ROC) analysis for biomarker studies. By considering sensitivity and specificity, the ability of a potential biomarker to discriminate between two patient groups can be better investigated. Furthermore, the interrelation between abundance variability and fold changes is also considered in statistical test theory. For example, the t-test is a generally accepted standard method to assess reliable biomarker candidates on the basis of group variances and the difference in group means. Nevertheless, the RCV-based assessment suggested here may be more intuitive and better interpretable for most researchers.

## Conclusions

We investigated whether high variability in CSF proteins may be a reason that only a limited number of protein biomarker candidates could be validated. In conclusion, our results indicate that high intra- and interindividual variability of a protein might lead to the identification of unsuitable biomarker candidates for multiple diseases. Thus, natural variability has a considerable impact on CSF protein biomarker research and must be taken into account in order to generate relevant results. The degree of variability in protein abundance is strongly protein dependent. Therefore, the variability should not be estimated over all proteins but instead calculated for each protein specifically. To this end, we recommend calculating the analytical variability, CV_a_, as well as the intraindividual variability, CV_t_, and interindividual variability, CV_g_, for each protein. Then, to assess whether the change in protein abundance between experimental groups might be an unexpectedly high change indicating biological relevance, we further recommend calculating RCV_g_ and RCV_t_, depending on the study design.

## Supporting information

S1 Table Neuropsychological characteristics of all participants and the representative study group analyzed in the present study. The analyzed representative study group reflects the total study group in terms of age and neuropsychological characteristics (e.g., Beck Depression Inventory (BDI) and Mini-Mental State Examination (MMSE)).
(DOCX)

S2 Table Blood and routine laboratory analysis for all participants and the representative study group analyzed in the present study. For all participants, clinically important blood parameters (e.g., red and white blood cell count, electrolyte concentrations and function parameters) were determined to ensure a neurologically healthy control group. The representative study group analyzed in the present study reflects the total study groups in terms of their blood analysis.
(DOCX)

S3 Table Coefficient of variation (CV) and reference change value (RCV) of the top 50 cerebrospinal fluid (CSF) core proteins in terms of label-free quantification (LFQ) intensity. The top 50 proteins of all samples were defined by their mean LFQ intensity. This table shows their protein names, UniProt ID, and intra- (CV_t_) and interindividual (CV_g_) coefficient.
(DOCX)

S1 File CSF protein list (223). All listed proteins were identified with at least one unique peptide in 100% of the samples. The file provides information regarding protein IDs, protein names, gene names, numbers of unique peptides, and LFQ intensities, as well as CV_t_, CV_g_, RCV_t_ and RCV_g_, for every protein.
(XLSX)

S2 File CSF protein list (610). All listed proteins were identified with at least two unique peptides in at least one sample. The file provides information regarding protein IDs, protein names, gene names, numbers of unique peptides and LFQ intensities for every sample.
(XLSX)

S3 File CSF core proteome (216). All listed proteins were identified with at least two unique peptides in 100% of the samples. The file provides information regarding protein IDs, protein names, gene names, numbers of unique peptides, and LFQ intensities, as well as CV_t_, CV_g_, RCV_t_ and RCV_g_, for every protein.
(XLSX)

S4 File CSF compositional proteins in healthy human individuals. Merging of the strict protein list (S1 File) and the extended table of proteins provided by *Hühmer et al*. and *Stoop et al*. The file provides information regarding protein IDs and protein names, as well as which proteins were listed by Hühmer *et al*. or *Stoop et al*. and were simultaneously identified in our samples.
(XLSX)

S1 Fig CV_t_ box plots. This graph shows the log2 CV_t_ values for every subject depending on the subject’s age.
(TIF)
