# Supplementary material for: Protein variability in cerebrospinal fluid and its possible implications for neurological protein biomarker research
Source: PLoS One. 2018 Nov 29;13(11):e0206478. doi: 10.1371/journal.pone.0206478 (PMC6264484; doi:10.1371/journal.pone.0206478)
Supplement: S1 Table — The analyzed representative study group reflects the total study group in terms of age and neuropsychological characteristics (e.g., Beck Depression Inventory (BDI) and Mini-Mental State Examination (MMSE)). (DOCX) [file pone.0206478.s001.docx]

**S1 Table. Neuropsychological characteristics of all participants and the representative study group analyzed in the present study.**

|  | **Standard value** | **Total** | | | | | **Non-study group** | | | | | **Study group** | | | | |
| --- | --- | --- | --- | --- | --- | --- | --- | --- | --- | --- | --- | --- | --- | --- | --- | --- |
|  |  | **N** | **Mean** | **SD*** | **CV*** | **Median** | **N** | **Mean** | **SD*** | **CV*** | **Median** | **N** | **Mean** | **SD*** | **CV*** | **Median** |
| Age [years] | 40-85 | 90 | 66.9 | 7.0 | 10.5% | 68.0 | 78 | 66.6 | 7.3 | 10.9% | 67.0 | 12 | 69.1 | 4.7 | 6.7% | 69.0 |
| NMS Quest total [points] | rating scale | 90 | 3.8 | 2.7 | 70.5% | 3.0 | 78 | 3.8 | 2.7 | 70.7% | 3.0 | 12 | 4.3 | 2.9 | 67.7% | 4.0 |
| NMS Quest sum [points] | rating scale | 90 | 0.1 | 0.1 | 70.7% | 0.1 | 78 | 0.1 | 0.1 | 70.9% | 0.1 | 12 | 0.1 | 0.1 | 68.4% | 0.1 |
| UPDRS total [points] | rating scale | 90 | 2.3 | 3.4 | 144.8% | 1.0 | 78 | 2.3 | 3.5 | 150.0% | 1.0 | 12 | 2.2 | 2.2 | 99.6% | 2.0 |
| MDS UPDRS total [points] | rating scale | 90 | 5.7 | 5.9 | 104.9% | 4.0 | 78 | 5.6 | 6.1 | 108.9% | 3.5 | 12 | 6.3 | 5.0 | 79.8% | 5.5 |
| BDI total [points] | 0-8 | 90 | 3.5 | 4.1 | 117.7% | 2.0 | 78 | 3.2 | 4.0 | 122.5% | 2.0 | 12 | 5.0 | 4.5 | 90.2% | 3.1 |
| MMSE total [points] | 24 -30 | 90 | 28.8 | 1.3 | 4.3% | 29.0 | 78 | 28.8 | 1.2 | 4.1% | 29.0 | 12 | 28.3 | 1.5 | 5.3% | 29.0 |
| Clock test: [points] | <2 | 90 | 1.4 | 0.7 | 51.5% | 1.0 | 78 | 1.4 | 0.7 | 52.6% | 1.0 | 12 | 1.3 | 0.6 | 43.3% | 1.0 |

The analyzed representative study group reflects the total study group in terms of age and neuropsychological characteristics (e.g., Beck Depression Inventory (BDI) and Mini-Mental State Examination (MMSE)).
