# Supplementary material for: Protein variability in cerebrospinal fluid and its possible implications for neurological protein biomarker research
Source: PLoS One. 2018 Nov 29;13(11):e0206478. doi: 10.1371/journal.pone.0206478 (PMC6264484; doi:10.1371/journal.pone.0206478)
Supplement: S2 Table — For all participants, clinically important blood parameters (e.g., red and white blood cell count, electrolyte concentrations and function parameters) were determined to ensure a neurologically healthy control group. The representative study group analyzed in the present study reflects the total study groups in terms of their blood analysis. (DOCX) [file pone.0206478.s002.docx]

**S2 Table. Blood and routine laboratory analysis for all participants and the representative study group analyzed in the present study**.

| **Blood parameter** | **Standard value** | **Total** | | | | | **Non-study group** | | | | | **Study group** | | | | | |
| --- | --- | --- | --- | --- | --- | --- | --- | --- | --- | --- | --- | --- | --- | --- | --- | --- | --- |
|  |  | **N** | **Mean** | **SD*** | **CV*** | **Median** | **N** | **Mean** | **SD*** | **CV*** | **Median** | **N** | **Mean** | **SD*** | **CV*** | **Median** |  |
| Red blood cell count [cells/pL] | 4,8-5,9(m) 4,3-5,2(f) | 90 | 4.8 | 0.7 | 15.5% | 4.8 | 78 | 4.8 | 0.8 | 16.3% | 4.8 | 12 | 4.7 | 0.3 | 7.1% | 4.7 |  |
| Hemoglobin [g/dL] | 13-17(m), 12-15(f) | 90 | 14.5 | 2.3 | 15.9% | 14.5 | 78 | 14.6 | 2.5 | 16.9% | 14.4 | 12 | 14.3 | 0.8 | 6.4% | 14.4 |  |
| Hematocrit [%] | 40-52(m), 36-47(f) | 90 | 42.4 | 3.4 | 8.0% | 42.3 | 78 | 42.4 | 3.4 | 8.1% | 42.3 | 12 | 42.3 | 2.2 | 11.7% | 42.4 |  |
| Mean corpuscular hemoglobin concentration (MCHC) [g/dL] | 30-35 | 90 | 34.2 | 3.5 | 10.3% | 34.0 | 78 | 34.3 | 3.8 | 11.0% | 34.1 | 12 | 33.9 | 0.6 | 8.6% | 33.8 |  |
| Mean corpuscular volume (MCV) [fL] | 80-100 | 90 | 89.1 | 6.9 | 7.7% | 89.2 | 78 | 88.8 | 7.1 | 8.0% | 88.9 | 12 | 91.2 | 3.6 | 9.2% | 92.3 |  |
| White blood cells (WBC)[10^9/L] | 4.0-10.0 | 90 | 6.0 | 2.1 | 34.6% | 5.6 | 78 | 6.0 | 2.2 | 36.2% | 5.7 | 12 | 5.8 | 1.0 | 22.4% | 5.3 |  |
| Mean corpuscular hemoglobin (MCH) [fmol/cell] | 0.4-0.5 | 90 | 31.2 | 15.0 | 48.1% | 30.3 | 78 | 31.3 | 16.1 | 51.6% | 30.3 | 12 | 30.9 | 1.1 | 4.9% | 31.3 |  |
| Platelets [10^9/L] | 150-400 | 90 | 230.1 | 58.6 | 25.5% | 228.0 | 78 | 230.2 | 59.7 | 26.0% | 228.0 | 12 | 229.6 | 47.5 | 17.0% | 236.3 |  |
| Total calcium: 2-2.6 mmol/L | 2.0-2.6 | 90 | 2.4 | 0.2 | 7.4% | 2.4 | 78 | 2.4 | 0.2 | 7.6% | 2.4 | 12 | 2.4 | 0.1 | 6.0% | 2.4 |  |
| Potassium: 3.5-5 mmol/L | 3.5-5.0 | 90 | 4.8 | 8.4 | 174.5% | 4.3 | 78 | 4.9 | 9.1 | 184.1% | 4.3 | 12 | 4.3 | 0.2 | 14.9% | 4.3 |  |
| Sodium: 135-145 mmol/L | 135-145 | 90 | 140.1 | 8.6 | 6.2% | 141.0 | 78 | 140.1 | 9.2 | 6.6% | 141.0 | 12 | 140.2 | 2.6 | 2.2% | 140.3 |  |
| Uric acid: 0.18-0.48 mmol/L | 0.18-0.48 | 90 | 5.7 | 1.8 | 31.7% | 5.5 | 78 | 5.7 | 1.8 | 31.8% | 5.5 | 12 | 5.6 | 1.6 | 20.4% | 5.0 |  |
| Urea: 1.2-3 mmol/L | 1.2-3.0 | 90 | 33.5 | 8.6 | 25.7% | 33.0 | 78 | 34.0 | 8.7 | 25.7% | 33.0 | 12 | 30.7 | 6.1 | 32.4% | 30.5 |  |
| Creatinine: 0.8-1.3 mg/dL | 0.8-1.3 | 90 | 1.4 | 6.2 | 432.0% | 0.9 | 78 | 1.5 | 6.6 | 437.2% | 0.9 | 12 | 0.9 | 0.2 | 12.0% | 0.9 |  |
| Alkaline phosphatase: 50-100 U/L | 50 - 100 | 90 | 68.2 | 16.9 | 24.8% | 66.0 | 78 | 69.3 | 17.0 | 24.6% | 67.0 | 12 | 60.9 | 12.8 | 19.6% | 59.3 |  |
| Gamma glutamyl transferase: 6-50 U/L | 6.0 -50 | 90 | 31.1 | 24.5 | 78.8% | 23.0 | 78 | 30.5 | 25.1 | 82.0% | 23.0 | 12 | 34.7 | 19.4 | 19.6% | 27.2 |  |
| Aspartate aminotransferase (AST): 5-30 U/L | 5.0 -30 | 90 | 26.5 | 11.0 | 41.6% | 25.0 | 78 | 26.3 | 11.4 | 43.5% | 24.4 | 12 | 27.8 | 5.4 | 19.7% | 26.6 |  |
| Alanine aminotransferase (ALT): 5-30 U/L | 5.0 -30 | 90 | 25.5 | 17.8 | 69.7% | 22.0 | 78 | 25.8 | 18.7 | 72.4% | 21.8 | 12 | 23.9 | 8.8 | 31.1% | 21.3 |  |
| Direct bilirubin: 0-6 µmol/L | 0- 6 | 90 | 0.6 | 0.3 | 43.2% | 0.6 | 78 | 0.6 | 0.3 | 42.5% | 0.6 | 12 | 0.7 | 0.3 | 34.1% | 0.6 |  |
| Creatine kinase: 25-200 U/L | 25 -200 | 90 | 132.0 | 81.4 | 61.7% | 116.0 | 78 | 129.5 | 71.7 | 55.4% | 114.5 | 12 | 148.3 | 85.3 | 35.9% | 134.7 |  |
| Total cholesterol: 3-5.5 mmol/L | 3.5 -5.0 | 90 | 233.3 | 41.1 | 17.6% | 233.0 | 78 | 231.6 | 41.3 | 17.9% | 230.0 | 12 | 244.4 | 32.2 | 17.8% | 235.0 |  |
| Triglycerides [mg/dL] | 50-150 | 90 | 124.2 | 81.2 | 65.4% | 108.5 | 78 | 124.4 | 85.6 | 68.8% | 106.5 | 12 | 123.1 | 36.7 | 41.3% | 117.7 |  |
| Thyroid-stimulating hormone (TSH) [mIU/L] | 0.5-5.0 | 90 | 1.7 | 1.0 | 59.3% | 1.6 | 78 | 1.7 | 1.0 | 61.6% | 1.6 | 12 | 1.7 | 0.5 | 41.1% | 1.5 |  |

For all participants, clinically important blood parameters (e.g., red and white blood cell count, electrolyte concentrations and function parameters) were determined to ensure a neurologically healthy control group. The representative study group analyzed in the present study reflects the total study groups in terms of their blood analysis.
