# Supplementary material for: Protein variability in cerebrospinal fluid and its possible implications for neurological protein biomarker research
Source: PLoS One. 2018 Nov 29;13(11):e0206478. doi: 10.1371/journal.pone.0206478 (PMC6264484; doi:10.1371/journal.pone.0206478)
Supplement: S3 Table — The top 50 proteins of all samples were defined by their mean LFQ intensity. This table shows their protein names, UniProt ID, and intra- (CVt) and interindividual (CVg) coefficient. (DOCX) [file pone.0206478.s003.docx]

**S3 Table. Coefficient of variation (CV) and reference change value (RCV) of the top 50 cerebrospinal fluid (CSF) core proteins in terms of label-free quantification (LFQ) intensity.**

| **Protein IDs** | **Protein name** | **CV_t_/CV_g_**  **[%]** | **RCV_t_/RCV_g_**  **[%]** | **Protein IDs** | **Protein name** | **CV_t_/CV_g_**  **[%]** | **RCV_t_/RCV_g_**  **[%]** | **Protein IDs** | **Protein name** | **CV_t_/CV_g_**  **[%]** | **RCV_t_/RCV_g_**  **[%]** | **Protein IDs** | **Protein name** | **CV_t_/CV_g_**  **[%]** | **RCV_t_/RCV_g_**  **[%]** |
| --- | --- | --- | --- | --- | --- | --- | --- | --- | --- | --- | --- | --- | --- | --- | --- |
| P02768 | Serum albumin | 6.9 / 21.0 | 19.0 / 58.3 | P02774 | Vitamin D-binding protein | 5.5 / 22.6 | 15.2 / 62.7 | P10645 | Chromogranin-A | 7.3 / 341.5 | 20.2 / 115.1 | P23142 | Fibulin-1 | 3.8 / 10.6 | 10.5 / 29.5 |
| P02787 | Serotransferrin | 3.1 / 10.2 | 8.6 / 28.2 | P01023 | Alpha-2-macroglobulin | 4.9 / 10.6 | 13.5 / 29.3 | Q9UBP4 | Dickkopf-related protein 3 | 7.1 / 24.8 | 19.6 / 68.8 | P01008 | Antithrombin-III | 5.7 / 27.1 | 15.7 / 75.0 |
| P41222 | Prostaglandin-H2 D-isomerase | 6.6 / 13.1 | 18.3 / 36.4 | P05090 | Apolipoprotein D | 6.1 / 19.9 | 17.0 / 55.1 | P00738 | Haptoglobin | 10.2 / 101.5 | 28.2 / 281.4 | Q12805 | EGF-containing fibulin-like extracellular matrix protein 1 | 5.5 / 9.8 | 15.1 / 27.2 |
| P01857 | Ig gamma-1 chain C region | 8.0 / 34.7 | 22.1 / 96.3 | P02766 | Transthyretin | 10.7 / 19.0 | 29.5 / 52.8 | P02749 | Beta-2-glycoprotein 1 | 7.7 / 23.6 | 21.2 / 65.5 | P00751 | Complement factor B | 5.0 / 24.6 | 13.9 / 68.3 |
| P01009 | Alpha-1-antitrypsin | 7.1 / 35.2 | 19.6 / 97.4 | P02763 | Alpha-1-acid glycoprotein 1 | 11.0 / 36.1 | 30.4 / 100.0 | P05060 | Secretogranin-1 | 8.8 / 27.4 | 24.3 / 75.9 | P10451 | Osteopontin | 10.7 / 33.6 | 29.7 / 93.1 |
| P01024 | Complement C3 | 2.6 / 19.0 | 7.2 / 52.6 | P06396 | Gelsolin | 7.4 / 11.4 | 20.5 / 31.6 | P01011 | Alpha-1-antichymotrypsin | 4.5 / 17.5 | 12.4 / 48.4 | O00533 | Neural cell adhesion molecule L1-like protein | 6.3 / 32.2 | 17.5 / 89.1 |
| P10909 | Clusterin | 10.2 / 12.8 | 28.2 / 35.4 | P01876 | Ig alpha-1 chain C region | 10.2 / 58.1 | 28.4 / 161.0 | Q96KN2 | Beta-Ala-His dipeptidase | 4.3 / 14.5 | 11.8 / 40.1 | Q14515 | SPARC-like protein 1 | 3.8 / 18.0 | 10.5 / 50.0 |
| P02790 | Hemopexin | 5.3 / 19.8 | 14.6 / 55.0 | P01859 | Ig gamma-2 chain C region | 10.0 / 45.2 | 27.8 / 125.2 | Q92876 | Kallikrein-6 | 5.0 / 16.4 | 13.7 / 45.5 | P25311 | Zinc-alpha-2-glycoprotein | 4.5 / 23.5 | 12.6 / 65.1 |
| P01034 | Cystatin-C | 10.6 / 15.4 | 29.4 / 42.7 | P01019 | Angiotensinogen | 8.0 / 16.9 | 22.3 / 47.0 | P00747 | Plasminogen | 5.0 / 25.4 | 14.0 / 70.3 | Q92823 | Neuronal cell adhesion molecule | 5.7 / 29.5 | 15.8 / 81.9 |
| P0C0L5 | Complement C4-B | 3.8 / 25.9 | 10.4 / 71.8 | P36955 | Pigment epithelium-derived factor | 10.2 / 14.2 | 28.2 / 39.4 | Q13822 | Ectonucleotide pyrophosphatase | 5.6 / 14.7 | 15.5 / 40.9 | P06727 | Apolipoprotein A-IV | 11.3 / 33.1 | 31.4 / 91.7 |
| P01834 | Ig kappa chain C region | 10.4 / 31.6 | 28.9 / 87.7 | P02751 | Fibronectin | 4.6 / 13.6 | 12.8 / 37.6 | P01042 | Kininogen-1 | 8.0 / 26.3 | 22.1 / 73.0 | P05155 | Plasma protease C1 inhibitor | 7.1 / 15.2 | 19.7 / 42.2 |
| P02647 | Apolipoprotein A-I | 6.4 / 31.0 | 17.7 / 85.8 | P00450 | Ceruloplasmin | 6.3 / 25.0 | 17.4 / 69.3 | P02765 | Alpha-2-HS-glycoprotein | 5.5 / 29.7 | 15.3 / 82.3 |  |  |  |  |
| P02649 | Apolipoprotein E | 6.5 / 31.8 | 17.9 / 88.2 | P08603 | Complement factor H | 3.7 / 11.2 | 10.3 / 31.0 | P04217 | Alpha-1B-glycoprotein | 7.7 / 28.4 | 21.3 / 78.8 |  |  |  |  |

The top 50 proteins of all samples were defined by their mean LFQ intensity. This table shows their protein names, UniProt ID, and intra- (CV_t_) and interindividual (CV_g_) coefficient of variations, as well as their RCV.
