# Supplementary figures and images for: Protein variability in cerebrospinal fluid and its possible implications for neurological protein biomarker research
Source: PLoS One. 2018 Nov 29;13(11):e0206478. doi: 10.1371/journal.pone.0206478 (PMC6264484; doi:10.1371/journal.pone.0206478)

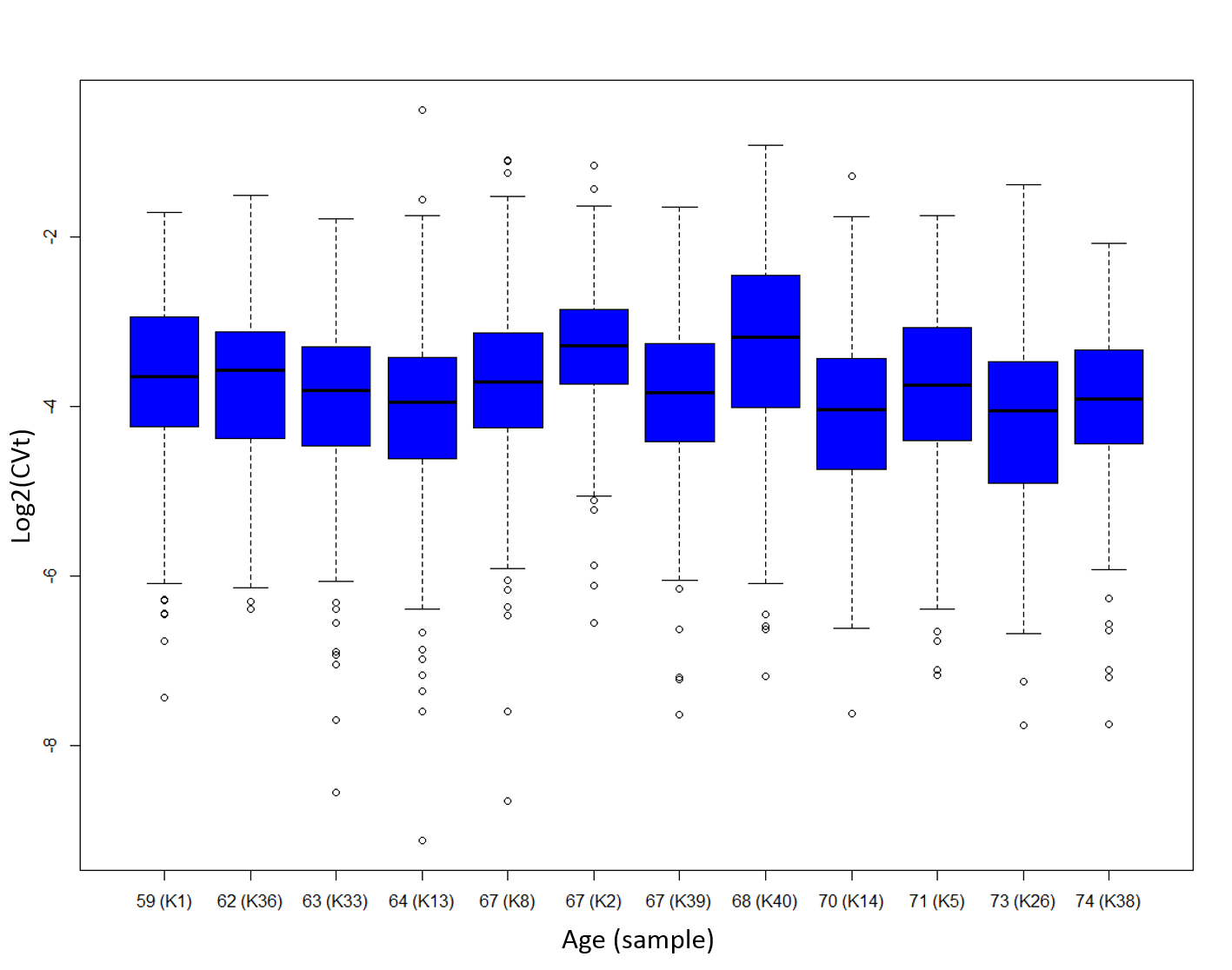

Supplement: S1 Fig — This graph shows the log2 CVt values for every subject depending on the subject’s age. (TIF) [file pone.0206478.s008.tif]
